# First person – Jessica Kabutomori

**DOI:** 10.1242/bio.054981

**Published:** 2020-08-14

## Abstract

First Person is a series of interviews with the first authors of a selection of papers published in Biology Open, helping early-career researchers promote themselves alongside their papers. Jessica Kabutomori is first author on ‘Water transport mediated by murine urea transporters: implications for urine concentration mechanisms’, published in BIO. Jessica is a masters student in the lab of Raif Musa Aziz at the Department of Physiology and Biophysics, University of São Paulo, investigating water transport across murine urea transporters UT-A2, UT-A3 and UT-B, towards the goal of better understanding the role(s) of these proteins in the kidney.


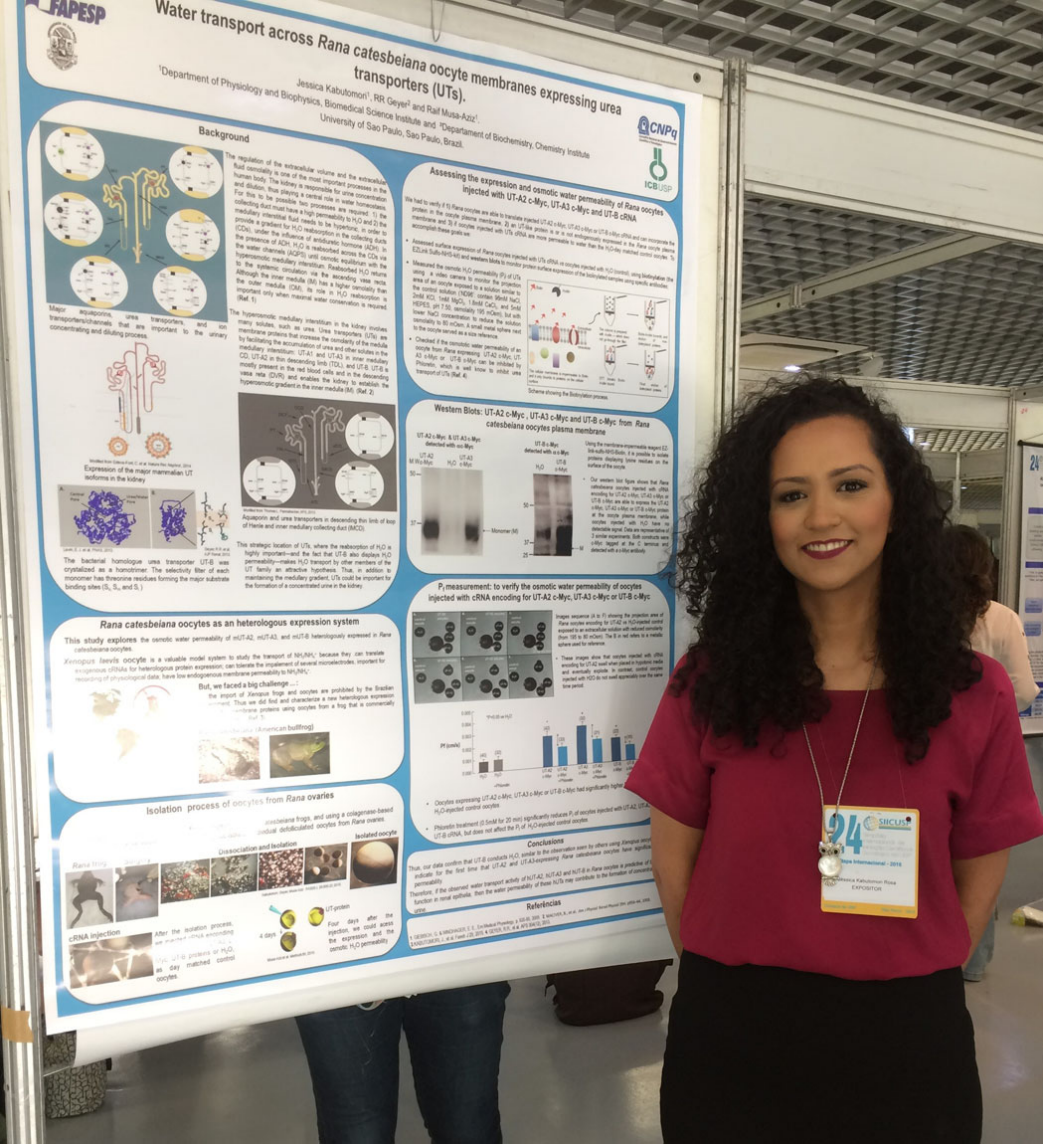


**Jessica Kabutomori Rosa**

**What is your scientific background and the general focus of your lab?**

For the past year, I have been working in the laboratory of Dr. Raif Musa-Aziz, where we study the water, urea and NH_3_ transport properties of membrane proteins heterologously expressed in *Lithobates catesbeianus* oocytes.

**How do you explain the main findings of your paper to non-scientific family and friends?**

I start by telling them how there are proteins in membranes of kidney cells that can transport urea - the chief nitrogenous end product of the metabolic breakdown of proteins, which are known as urea transporters (UTs) and are essential for concentrating the urine to minimize water loss by the body. Different parts of the kidney express different UTs and some of the known UTs include UT-B, UT-A2 and UT-A3. Interestingly, it has been shown that UT-B can also transport water. This observation led our laboratory to hypothesize that other UTs also transport water. If true, such a role could reshape our understanding of how the kidney concentrates (or dilutes) urine. To investigate this possibility, we used *Lithobates catesbeianus* oocytes, which are frog eggs that when injected with genetic material can express membrane proteins on the surface of the cells, and monitored urea and water transport activities. In our most recent study, we were able to demonstrate that in addition to transporting urea, UT-B, UT-A2 and UT-A3 are also capable of transporting water.

**What are the potential implications of these results for your field of research?**

Our observation that UT-B, UT-A2 and UT-A3 can transport both urea and water provides new insights into the role of these proteins for urine concentration and regulation of renal water excretion. To better understand the transport properties of this family of proteins, we are currently working on elucidating the molecular mechanisms underlying the substrate specificity. This will not only improve our understanding of the physiological roles of UTs but could also lead to the development of novel UT-targeted diuretics.

**What has surprised you the most while conducting your research?**

For many years, UTs have been the subject of many renal physiology studies. So I guess what surprised me the most was that even in 2020 we were able to demonstrate a new role for these proteins. In the present manuscript, we developed a model that explains how water transport across the UTs can contribute to the urinary concentrating mechanisms.

“For many years, UTs have been the subject of many renal physiology studies. … what surprised me the most was that even in 2020 we were able to demonstrate a new role for these proteins.”

**What, in your opinion, are some of the greatest achievements in your field and how has this influenced your research?**

The previous observations from the laboratories of Dr. Alan Verkman and Dr. Walter Boron, showing that UT-B could transport water, led our laboratory to hypothesize that other UTs may also exhibit such a function. In addition to these two studies, the strategic location of the different UT isoforms had a strong influence on our study and, consequently, our proposed model.

**Figure:**
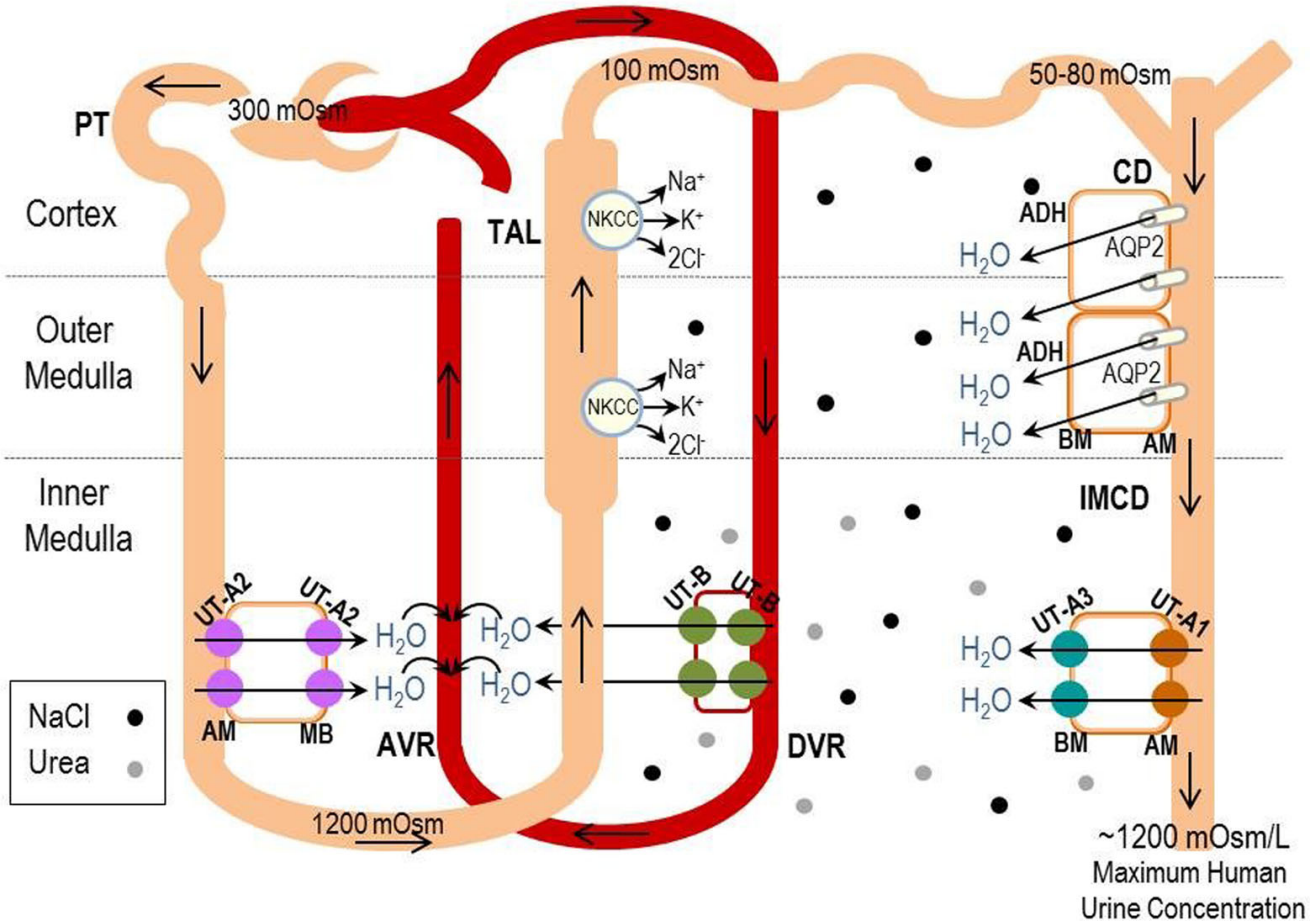
**Illustrated model of potential UT-mediated water transport contributions in the human renal inner medulla.**

**What changes do you think could improve professional lives of early-career scientists?**

That is a really difficult question to answer. As a graduate student, I see how hard the professors work to keep their labs up and running. In this sense, I think more funding needs to be set aside for early-stage scientists so that they can test new ideas and push their science forward.

**What's next for you?**

There is evidence in the literature that UT-B can transport ammonia (NH_3_). Thus, my colleague Neydiana Belize-Lopes and I, under the supervision of Dr Musa-Aziz, are going to explore whether other UTs can also perform NH_3_ transport. After that, the next step is to investigate, through site-directed mutagenesis in the monomeric urea pore, the molecular mechanisms by which UTs transport water and NH_3_. We believe that UTs are an important nexus for integrating the excretion of nitrogenous wastes, water and acid. In addition, I am investigating the influence of *N*-glycosylation on UT expression and function. In terms of my future goals as a young scientist, I intend on finishing my master's degree and then apply to a PhD program, where I will continue pursuing my career in science.
